# Developing Future Leaders in Health Assessment Research: Evaluation of interRAI’s inSPIRe Program

**DOI:** 10.1177/08404704251388358

**Published:** 2025-11-06

**Authors:** Julie Weir, Darly Dash, Danelle Kenny, Joanna Hikaka, Yassine Benhajali, Zain Pasat, Andrew P. Costa, Luke Andrew (LA) Turcotte, John Hirdes

**Affiliations:** 1 3427 University of New Brunswick, Fredericton, New Brunswick, Canada; 2 10068 Horizon Health Network, Moncton, New Brunswick, Canada; 3 3710 McMaster University, Hamilton, Ontario, Canada; 4 1974 University of Queensland, Brisbane, Queensland, Australia; 5 1415 University of Auckland, Auckland, New Zealand; 6 5620 The Douglas Research Center, McGill University, Montreal, Quebec, Canada; 7 Centre for Integrated Care, St. Joseph’s Health System, Hamilton, Ontario, Canada; 8 The Research Institute of St. Joseph’s Hamilton, Hamilton, Ontario, Canada; 9 7497 Brock University, St. Catharines, Ontario, Canada; 10 8430 University of Waterloo, Waterloo, Ontario, Canada

## Abstract

This article reports on the fourth interRAI Summer Program of International Research (inSPIRe), an intensive capacity-building initiative with a structured program, hosted at McMaster University in July 2024. Twenty-four delegates from 14 countries attended, representing diverse backgrounds in research, clinical practice, policy, and health informatics. The inSPIRe initiative aimed to foster understanding of interRAI’s assessment systems, develop methodological skills, establish mentorship relationships, create opportunities for contribution to the interRAI consortium, and initiate global collaborations. All participants reported that the program met or exceeded their expectations, with significant benefits including access to comprehensive international datasets, engagement with experienced mentors, and effective knowledge translation between research and practice. Regional adaptations of the program have already emerged, demonstrating its scalability and impact beyond the initial intensive experience. The inSPIRe program represents an effective and flexible model for building global health services leadership and research capacity and capability, applicable internationally.

## Background

The interRAI network (international Resident Assessment Instruments) is a global, collaborative network of researchers and practitioners committed to delivering evidence-informed, high-quality healthcare and health systems design, which subsequently informs efficient clinical practice and policy decision-making. From its original purpose to design a comprehensive assessment component in response to a key priority of the Omnibus Budget Reconciliation Act of 1987 enacted by the United States Congress,^1^ interRAI has evolved into a comprehensive, robust, and reliable system of assessment instruments that facilitate care coordination, resource allocation, monitoring, and evaluation functions of health systems worldwide.^2,3^ To sustain growth as a leading health services research organization, interRAI recognizes the need to invest not only in refining assessment instruments but also in fostering the expansion of their international network of fellows.

interRAI fellows engage in research, education, development, and implementation of interRAI assessment tools and health leadership worldwide.^4^ To receive an appointment to join the consortium as a fellow, an active interRAI fellow is required to make a nomination, with supporting documentation to the interRAI Board. The interRAI Board then determines the appropriateness of the candidate and sends out invitations to those chosen. Once engaged with the consortium, fellows benefit from access to the assessment suite across policy, industry practice, and research settings, accomplishing strides in their respective fields.

The interRAI Summer Program of International Research (inSPIRe) represents one of interRAI’s strategic initiatives designed to achieve network growth, build capacity, revitalization, and sustainability. Our article reports on the experience and outcomes of the fourth iteration of inSPIRe, hosted at McMaster University in Hamilton, Canada, by Drs. Andrew Costa and Luke Turcotte from July 12-22, 2024. We describe and evaluate inSPIRe as a translational example of developing leadership and sustainability in large programs, networks, and organizations. We also detail the program components to enable uptake, adaption, and implementation in other international contexts.

### Program Structure and Objectives

Entry to the inSPIRe program was via expression of interest after nomination by an interRAI fellow. The program targeted doctoral students, post-doctoral fellows, early career researchers, or high-level health practitioners and leaders with backgrounds relevant to interRAI’s mission. Successful applicants were then invited to a week-long in-person meeting.

The stated objectives of the inSPIRe program were for delegates to:

1. Gain an understanding of the interRAI network and related research;
2. Develop skills and capacity related to interRAI research methods and areas of focus;
3. Develop relationships with and benefit from mentorship offered by interRAI fellows;
4. Understand the opportunities to contribute to the interRAI consortium as research fellows or collaborators; and
5. Initiate global collaborators.

An intensive curriculum was designed to allow delegates to achieve these objectives. Each morning included targeted seminars led by interRAI fellows and senior interRAI staff, including Board Directors, followed by delegate presentations linking their interests, goals, and work to interRAI’s agenda. The didactic strategy employed was to progressively orient the delegates from foundational concepts and structures that define the interRAI collaborative toward key areas of scientific development and collaborative opportunities. The 5-day schedule exposed delegates to interRAI concepts, including the interRAI suite and community organization; foundational analytic concepts central to analyzing interRAI data; core methods of clinical decision support and performance measurement; new areas of translational applied science; and foundational concepts and examples for international collaboration. Current interRAI fellows interacted with delegates to illustrate both the technical and collaborative social context that defines the interRAI intellectual and social tradition.

In the afternoons, delegates worked in assigned teams on dedicated projects. Surveys were distributed weeks prior to the event to gather focus areas (e.g., pediatrics, seniors, and mental health) and capacities (e.g., clinical, policy, implementation, analyses, and informatics). Delegates were assigned to teams based on their primary or secondary focus area and each team had an equal distribution of capacities necessary to succeed in project work. Each team was composed of 4-5 delegates who developed a research question and methodology to investigate their question, conducted interim analyses drawing on the interRAI de-identified data archives provided by the University of Waterloo’s interRAI secure data server, and contextualized the research question and interim or anticipated findings in relevant practice and/or policy settings. Multiple interRAI mentors attended each session and circulated through groups, sharing ideas and expertise. Team progress was reported daily to delegates and mentors to stimulate team discussions, keep work on track, and refine ideas to reach presentation quality and meet scientific standards. Opportunities to contribute to interRAI were not only discussed but also enacted through the development of research suitable for presentation and publication.

In recognition of the central role of collaboration to the sustainability of networks like interRAI, the intensive schedule was complemented with informal interactions during evenings and weekends. Field trips to local landmarks fostered meaningful connections, and evening meals allowed delegates time to network outside the classroom/workspace. Unique food item sharing (“World Food Fair”), musical playlists, and team sport activities (pickleball) were designed to acknowledge cultural contexts and build camaraderie among delegates to carry forward into their careers. All activities reflected the concepts which are central to interRAI’s collegial culture of intense work and deliberations, within a supportive team-first context.

## Methodology

Following inSPIRe, organizers drafted a post-meeting evaluation survey using Qualtrics to assess the experiences of all 24 participants and the extent to which the program met its pre-defined objectives. The survey consisted of 30 questions combining multiple-choice and free-text responses, covering motivation, experiences relating to objectives, perceived benefits, areas for improvement, group-work experience, and intentions for future engagement with interRAI. We reported categorical responses using absolute (count) and relative (frequency) values. We coded free-text responses for each question using an iterative approach to develop subthemes for each program objective.^5^ The authorial team discussed subthemes until consensus was reached.

## Results

### Delegate Demographics and Experience

**Figure 1.**
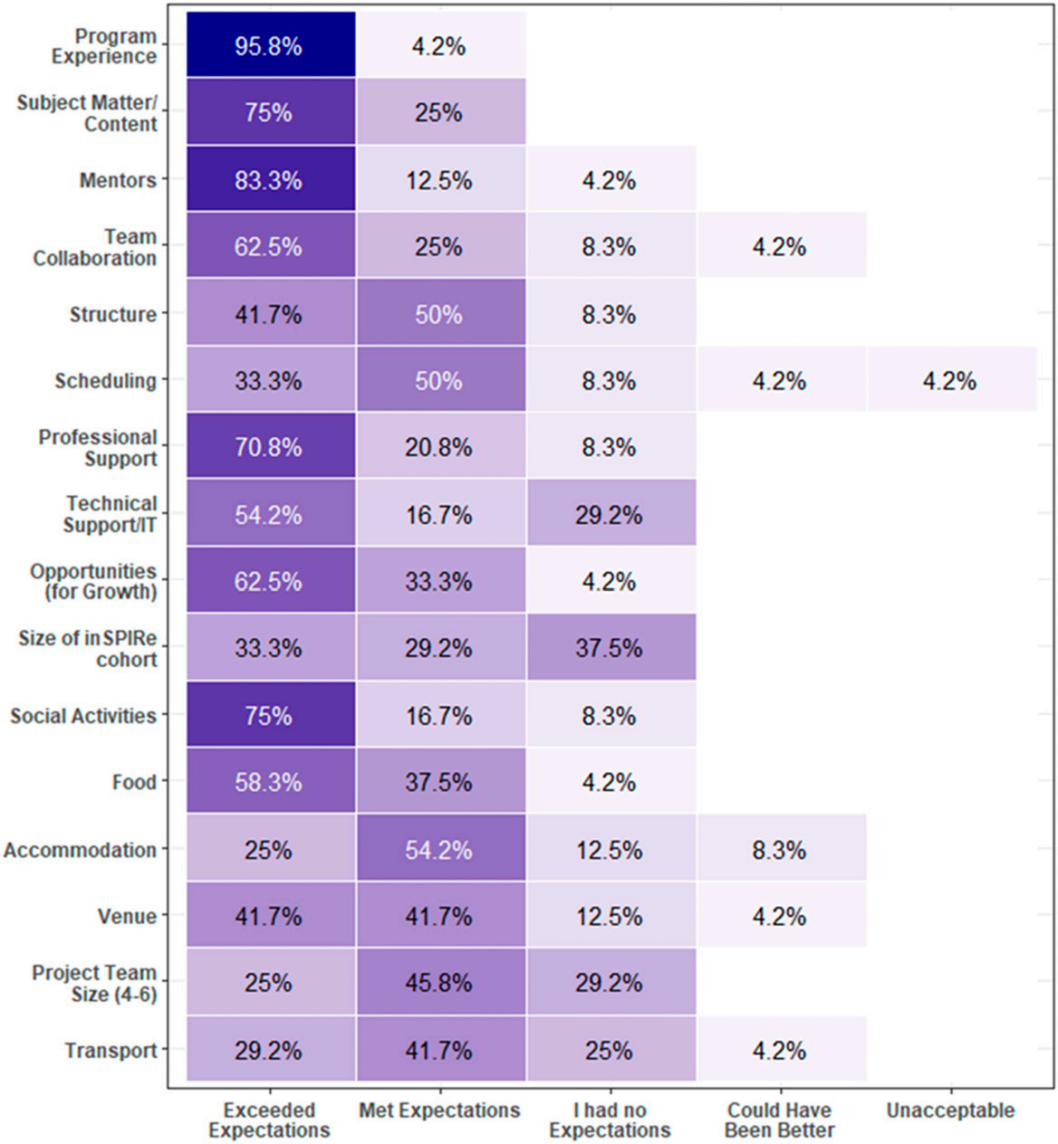
Delegate Experiences With inSPIRe 2024

All 24 delegates completed the evaluation survey. Delegates travelled from 14 countries: Canada, the United States, Australia, New Zealand, Hong Kong, Finland, Belgium, the United Kingdom, Poland, the Netherlands, Costa Rica, Italy, Switzerland, and South Korea. The most common primary motivations for participating in inSPIRe were to learn more about interRAI (42%), to increase engagement with interRAI (29%), and to pursue international collaboration (21%). The program experience met or exceeded expectations for all delegates (Figure 1). All indicated they would likely participate in a similar program again and would recommend inSPIRe to other researchers.

### Understanding interRAI Opportunity and Building Capacity

The inSPIRe program delivered comprehensive learning experiences that significantly expanded delegates’ understanding of interRAI. Delegates gained deep insights into the global applications of interRAI instruments, methodological approaches, health systems design, and potential for evidence-based decision-making in healthcare. Some of the highlights featured exposure to global perspectives on interRAI research through the interRAI fellow-led lectures and presentations, access to hands-on data analysis opportunities, and practical skills in research methodologies and data interpretation. These experiences culminated in a deeper understanding of interRAI’s role in public policy and healthcare. Delegates appreciated the program’s intensive, focused approach, allowing rapid knowledge acquisition across diverse healthcare research contexts. The learning environment accommodated varying technical backgrounds, providing foundational and advanced insights into interRAI research. These activities accurately reflect the program objectives of understanding interRAI and developing research skills using interRAI data.

### Global Mentorship and Future Opportunities

The networking dimension emerged as perhaps the most transformative aspect of the program. Delegates developed strong international professional relationships with other delegates and interRAI mentors, long-lasting collaborative partnerships across global research communities, and opportunities for future joint research and international health leadership opportunities.

These networking outcomes were achieved as a direct result of the efforts of the program organizers to prioritize opportunities for meaningful interactions through social activities and team-building events, structured mentorship experiences, interdisciplinary team projects, and shared cultural experiences. Delegates expressed clear intentions to continue their collaborations, including plans to collaborate on future research projects, implement interRAI tools in local healthcare settings, develop national efforts to promote interRAI, and organize training programs to educate others about interRAI tools. These opportunities position the inSPIRe program as a catalyst for ongoing global mentorship and collaborations in healthcare research and reflect all five of the program outcomes, engineering sustainability of the interRAI consortium as a global leader in health services research.

### Global Research Work

A cornerstone of the inSPIRe program was the collaborative research component, where five interdisciplinary teams developed and pursued unique research questions using interRAI data. Key highlights included a data-driven and evidence-based approach to research that allowed for team-driven, autonomous research experiences applying interRAI tools to real-world questions. These teams are at various stages of readiness for publication (Table 1). Each team worked intensively to formulate research questions addressing gaps in existing literature, demonstrating the program’s commitment to advancing interRAI research. The collaborative nature fostered creative problem-solving and innovative thinking across healthcare and research domains and allowed international connections to extend interRAI’s global network. This work aligns well with the program outcome to initiate global collaborations.

**Table 1.** inSPIRe 2024 Group Collaboration Outcomes

Team	Topic/title	Research objective	Datasets used	Status
Team 1	Mental Health/30 Day Readmission Risk among Mental Health Inpatients in Ontario	To identify risk factors of 30-day readmission among patients admitted to inpatient mental health beds	78,537 patient records from Resident Assessment Instrument-Mental Health (RAI-MH) in Ontario, Canada	Final draft of manuscript in preparation
Team 2	Care Needs and Self-sufficiency Assessment of Home Care Clients	To describe the correlation between subjective judgment of changes in self-sufficiency and measured changes in health status	70,369 community-dwelling older adults with an interRAI Home Care (HC) assessment between July 2021-Dec 2023	Published: *Pasat Z, Van Grootven B, Kooijmans E, Paulamäki J, Turcotte LA, Hirdes JP, Costa AP. Care Needs and Self-Sufficiency Assessment of Home Care Clients. Journal of the American Medical Directors Association. 2025 Jun 1;26(6):105572*
Team 3	Predictors of Caregiver Distress among Ontario Home Care Clients: A Comparative Analysis of 2002-2006 and 2018-2023 interRAI Home Care Datasets	Evaluate risk factors of caregiver distress over time	593,305 interRAI-HC 2018-2023 baseline assessments	Draft of manuscript in preparation
Team 4	Predictive Factors for Control Over Long-Term Care Admission Decisions: A Decision Tree Analysis	To derive and validate a predictive scale on the control over the decision to move into Long-Term Care (LTC)	13,650 LTC resident records from the interRAI Long-Term Care Facilities (LTCF) first assessments in Saskatchewan and New Brunswick, Canada	Final draft of manuscript in preparation
Team 5	Predictors of Change in Sleep Disturbance in Canadian Long-Term Care Facilities: A Longitudinal Analysis Based on interRAI Assessments	Identify predictors of change in sleep disturbances in Canadian long-term care facilities	21,294 interRAI LTCF records (4,645 assessments) from Saskatchewan, Canada, from July 1, 2019, to December 29, 2021	Published: *Wabe N, Geyskens L, Yi JY, Varratta E, Queirós AM, Turcotte LA, Costa A, Hirdes JP. Predictors of change in sleep disturbance in Canadian long-term care facilities: a longitudinal analysis based on interRAI assessments. European Geriatric Medicine. 2025 Sep 10:1-1*

### Benefits of the Program

#### Access to International Datasets

One of the most significant benefits of the inSPIRe program was access to comprehensive datasets. InSPIRe delegates worked with large datasets comprising standardized detailed health assessments across multiple care settings. Large sample sizes enabled robust statistical modelling, and the longitudinal nature of the data allowed for temporal analysis. This provided unique opportunities for comparative analysis and insights into cross-cultural healthcare patterns to understand system-level differences. Working with these datasets allowed delegates to develop research questions with global relevance while gaining practical experience in handling complex healthcare data. The collaborative approach to data analysis fostered the sharing of methodological techniques across different research traditions.

#### Mentor Engagement

Delegates highlighted that the positive outcomes experienced were made possible due to the strong engagement and involvement of mentors. These experienced interRAI fellows and associates brought their knowledge, skills, years of experience, and genuine interest in and commitment to both interRAI and inSPIRe’s objectives, which were central to the program’s success. Specifically, mentors provided domain expertise in relevant healthcare sectors, methodological and statistical guidance, contextual knowledge on interRAI assessments, network connections, and career development advice for all delegates, regardless of career stage or previous associations with interRAI. The mentors provided real-time face-to-face support in a way that was evidence-informed, backed by years of experience and encouraged healthy debate of opposing opinions. In this way, delegates witnessed firsthand the interaction style of interRAI fellows where there is no space for the hierarchical or authoritative approach.

#### Knowledge Translation

The program bridged research and practice by equipping participants to translate data into actionable insights for real-world healthcare settings. interRAI provided a common language for discussing healthcare assessment. Cross-jurisdictional and multi-disciplinary teams provided for learning about interRAI implementation strategies in real-world practice and policy contexts. Together these contributed to the capacity building of delegates, increasing the likelihood of implementing evidence-based practices in the future.

### Challenges and Areas for Improvement

Despite its many successes, the inSPIRe program faced several challenges that warrant consideration for future iterations. The program encountered technical and methodological barriers that impacted some delegates’ experiences. The varying levels of statistical and data analysis expertise among delegates created learning curve challenges for some while potentially slowing progress for others. The limited time frame for complex data analysis and interpretation placed pressure on teams to rapidly develop and execute research plans. Many groups faced technical issues accessing and managing large datasets, requiring creative problem-solving within the available timeframe. Throughout the program, teams face challenges balancing analytical rigour with limited time, sometimes requiring trade-offs in scope or methodology.

Although the multinational teams provided an opportunity for cross-country collaboration, significant challenges are presented by legal and regulatory limitations affecting data access and secondary use. The varying privacy laws across jurisdictions complicate data sharing and limit certain comparative analyses, especially data sharing across countries. While the “collect once, use many times” principle of interRAI supports efficiency, operationalization faces substantial barriers. Ethical considerations around secondary data use require careful navigation, particularly sensitive, health-related information. Administrative hurdles in accessing and linking datasets consumed valuable time during the already intensive program schedule.

Program structure considerations present additional challenges that affect the learning experience. While the intensive format enabled rapid progress, some delegates reported information overload. Balancing structured learning activities with self-directed teamwork creates challenges in meeting diverse learning needs and interests. Several delegates noted the need for more extensive pre-program preparation to maximize the value of in-person time. Ensuring appropriate follow-up mechanisms to sustain momentum after the program is concluded also requires support for long-term collaboration and project completion to support the community of practice.

### Future Opportunities

The inSPIRe program has created numerous possibilities for expansion and replication, further expanding the interRAI network while maintaining its reputation as a global leader in health services research. The inSPIRe cohort have continued to interact and engage with each other on social media and through collaborative projects such as this paper.

Over the months following the inSPIRe 2024 event, several participants have been invited to join the interRAI network as associate interRAI fellows and others continue to work collegially to advance the interRAI agenda globally. This integration into the formal interRAI community creates sustainable pathways for leadership, ongoing engagement, and expansion of the interRAI global network.

Third, the research projects initiated during inSPIRe have continued to develop after the program concluded, with some teams accepted for publication and others approaching submission of manuscripts, demonstrating the effectiveness of inSPIRe to create lasting research partnerships and outputs extending well beyond the initial intensive experience. Beyond formal research outcomes, delegates are positioned to become knowledge brokers, bringing interRAI expertise back to their home countries and institutions. The Australian interRAI community has already initiated the ASPiRe (Australian Summer Program of interRAI Research) program; a regional variation of the same program conducted in February 2025, employing a similar structure while adapting to local needs and constraints. This regional adaptation demonstrates opportunities for lessons learned and best practices to be refined and deployed across different settings, creating a scalable, internationally applicable, capacity-building model. This knowledge translation function amplifies the program’s impact by creating multiple hubs of interRAI expertise internationally across diverse geographic and healthcare contexts.

## Limitations

This article presents a post-program evaluation of the interRAI’s inSPIRe 2024 program. We were unable to evaluate the contributions of delegates from previous inSPIRe cohorts. While we detail the impacts of the 2024 cohort (e.g., manuscripts published or in progress and invitations to become associate research fellows), future work is warranted to continue monitoring the long-term effects of the program. The effectiveness of the interRAI approach is evident with the implementation of the interRAI assessments in 35 countries, a compendium of over 2,300 peer-reviewed research articles and books spanning decades of health services research, and interRAI’s involvement in high-level strategic and systems thinking working with governments and health policy leaders.^6^

## Conclusion

Our evaluation confirms inSPIRe’s pre-defined program objectives were achieved. The interRAI inSPIRe program demonstrates the value of intensive, collaborative approaches to building capacity in health services research, policy, and healthcare decision-making. By bringing together delegates and practitioners from diverse backgrounds and countries, inSPIRe provides a powerful platform for learning, research and leadership development, and network building, generating enormous capacity to expand the expertise, global reach, and success of the interRAI brand. Its reach has extended from humble beginnings in aged care to include additional populations and settings; however, their overarching goal to improve care specific to the population involved remains.^4^ Engagement and involvement of new healthcare leaders is central to the sustainability of interRAI and its mission.

InSPIRe’s success stems from its integrated model combining education, mentorship, research collaboration, and social engagement. Delegates gain technical knowledge and skills while building meaningful professional relationships and contributing to advancing interRAI’s international agenda. This approach to capacity building in health services research and health leadership is flexible and adaptable and should be considered in other healthcare research domains.

As interRAI’s global reach continues to expand, programs like inSPIRe will be instrumental in developing the next generation of leaders committed to advancing healthcare through evidence-based healthcare assessment. The collaborations emerging from the program suggest that its impact extends well beyond the intensive week-long experience, developing the next generation of interRAI fellows, and creating lasting contributions to healthcare assessment and quality improvement around the world.

## Data Availability

Access to anonymized data is available upon request to the corresponding author.*
